# Desmoplastic infantile astrocytoma with atypical phenotype, *PTEN* homozygous deletion and *BRAF V600E* mutation

**DOI:** 10.1186/s40478-022-01392-x

**Published:** 2022-06-20

**Authors:** Javier Megías, Teresa San-Miguel, Mirian Sánchez, Lara Navarro, Daniel Monleón, Silvia Calabuig-Fariñas, José Manuel Morales, Lisandra Muñoz-Hidalgo, Pedro Roldán, Miguel Cerdá-Nicolás, Concha López-Ginés

**Affiliations:** 1 Department of Pathology, Faculty of Medicine and Dentistry, University of Valencia, Avenida de Blasco Ibáñez, 15, 46010 Valencia, Spain; 2 Centro de Investigación Biomédica en Red en Cáncer (CIBERONC), Valencia, Spain; 3 Department of Neurosurgery, Clinic Hospital of Valencia, Valencia, Spain; 4 INCLIVA, Clinic Hospital of Valencia, Valencia, Spain

**Keywords:** Desmoplastic infantile astrocytoma, *PTEN*, *BRAF V600E*, Atypical

## Abstract

Desmoplastic infantile astrocytoma (DIA) is rare, cystic and solid tumor of infants usually found in superficial cerebral hemispheres. Although DIA is usually benign, uncommon cases bearing malignant histological and aggressive clinical features have been described in the literature. We report a newborn patient who was diagnosed with a DIA and died postresection. Pathologic examination revealed that the main part of the tumor had benign features, but the internal region showed areas with a more aggressive appearance, with higher-proliferative cells, anaplastic GFAP positive cells with cellular polymorphism, necrosis foci, vascular hyperplasia with endothelial proliferation and microtrombosis. Genetic study, performed in both regions of the tumor, showed a *BRAF V600E* mutation and a homozygous deletion in *PTEN*, without changes in other relevant genes like *EGFR*, *CDKN2A*, *TP53*, *NFKBIA*, *CDK4*, *MDM2* and *PDGFRA*. Although *PTEN* homozygous deletions are described in gliomas, the present case constitutes the first report of a *PTEN* mutation in a DIA, and this genetic feature may be related to the malignant behavior of a usually benign tumor. These genetic findings may point at the need of further and deeper genetic characterization of DIAs, in order to better understand the biology of this tumor and to obtain new prognostic approaches, a better clinical management and targeted therapies, especially in malignant cases of DIA.

## Introduction

Desmoplastic infantile astrocytoma (DIA) is a meningocerebral neuroepithelial tumor of infancy defined by a combination of distinctive clinicopathologic features [1]. DIA was first described as a meningocerebral astrocytoma attached to dura with desmoplastic reaction [2], and later was included in the World Health Organization defined as a desmoplastic cerebral astrocytoma of infancy [1]. DIA is considered a biologically benign neoplasm. The large majority of cases are usually diagnosed in the first two years of life [1, 3]. Despite the fact that DIA has been generally considered as a tumor of infants, it can also be seen in older patients. The non-infantile cases are rare, with few cases reported previously [3–12]. When surgical complete resection is achieved, it is followed by a favorable postoperative course. However, in some cases, atypical, aggressive, and multifocal variants of DIA have been described [11, 13–17].

Here, we report a rare case of DIA in a male newborn, with histological characteristics of malignancy, *BRAF V600E* mutation and *PTEN* homozygous deletion. Genetic studies of DIA are very scarce. Since its first description in 1984, only twelve works performed genetic search of DIA mutations, and most of them focused exclusively on the detection of the well characterized *BRAF V600E* mutation [10–12, 18–26]. To our knowledge, only eleven cases of DIA with this *BRAF* mutation have been described [10–12, 18–20, 23, 26], and mutations in *PTEN* have never been reported. Table 1 shows a summary of the existing literature of DIA, highlighting the publications with genetic studies (Table 1).

**Table 1:** Cases of DIA described in literature

References	Age	Sex	Genetics	*BRAF* mutation
Taratuto et al. [2]	6 months	Female	No	–
	6 months	Female	No	–
	6 months	Male	No	–
	1.5 month	Male	No	–
	7 months	Male	No	–
	9 months	Female	No	–
Chacko et al. [4]	7 years	Female	No	–
VandenBerg (9 cases) [27]	1.5 to 14 months	4 males/5 females	No	–
Rushing et al. [28]	6 months	Female	No	–
Al-Sarraj and Bridges [29]	8 months	Male	No	–
Prayson [30]	3 months	Male	No	–
Setty et al. [13]	4 months	Male	No	–
Mallucci et al. [5]	3.5 years	–	No	–
	3 months	–	No	–
	5 months	–	No	–
Sugiyama et al. [31]	4 months	Female	No	–
	2 months	Female	No	–
	5 months	Female	No	–
	4 months	Female	No	–
Kato et al. [6]	9 years	Male	No	–
Darwish et al. [14]	4 months	Male	No	–
Beppu et al. [32]	1 year	Male	No	–
Santhosh et al. [7]	11 years	Male	No	–
Tsuji et al. [33]	3 months	Male	No	–
Ulu et al. [3]	4 years	Female	No	–
Gu et al. [34]	1 month	Male	No	–
Uro-Coste et al. [8]	5 years	Male	No	–
Phi et al. [15]	7 months	Female	No	–
Rasalkar et al. [9]	18 years	Female	No	–
Al-Kharazi et al. [16]	3 months	Male	No	–
Gessi et al. [18]	2 months	Male	Yes	No
	2 years	Male	Yes	*BRAF V600E*
	3 months	Male	Yes	No
	8 months	Female	Yes	No
Karabagli et al. [10]	6 years	Male	Yes	*BRAF V600E*
Koelsche et al. [19]	4 months	Male	Yes	*BRAF V600E*
Abuharbid et al. [20]	11 months	Female	Yes	*BRAF V600E*
Greer et al. [21]	3 months	Female	Yes	No
	1 month	Male	Yes	No
	11 months	Female	Yes	No
Narayan et al. [17]	8 months	Female	No	–
Samkari et al. [35]	1.5 years	Male	No	–
Wang et al. [11]	3 months	Female	Yes	No
	7 months	Female	Yes	*BRAF V600E*
	11 years	Female	Yes	No
	6 years	Male	Yes	*BRAF V600E*
	4 months	Female	Yes	*BRAF V600E*
Chatterjee et al. [12]	10 years	Female	Yes	*BRAF V600E*
	10 years	Male	Yes	*BRAF V600E*
Naylor et al. [22]	3 months	Female	Yes	No
Van Tilburg et al. [23]	4.5 months	Male	Yes	*BRAF V600E*
Clarke et al. [24]	> 1 year	Female	Yes	No
	≤ 1 year	Female	Yes	No
	≤ 1 year	Male	Yes	No
Imperato et al. [25]	4 months	Female	Yes	No
	7 months	Male	Yes	No
	8 months	Male	Yes	No
	1 year	Female	Yes	No
	3 months	Male	Yes	No
	3 months	Female	Yes	No
	3 months	Male	Yes	No
	3 months	Female	Yes	No
	3 months	Male	Yes	No
	11 months	Female	Yes	No
	8 months	Female	Yes	No
	2 months	Female	Yes	No
Chiang et al. [26]	2 months	Female	Yes	*BRAF V600E*
	1 year	Female	Yes	No
	1 month	Female	Yes	No
Megías et al. (present case)	1 week	Male	Yes	*BRAF V600E*

Genetic studies and presence of *BRAF* mutation

## Case presentation

**Fig. 1:**
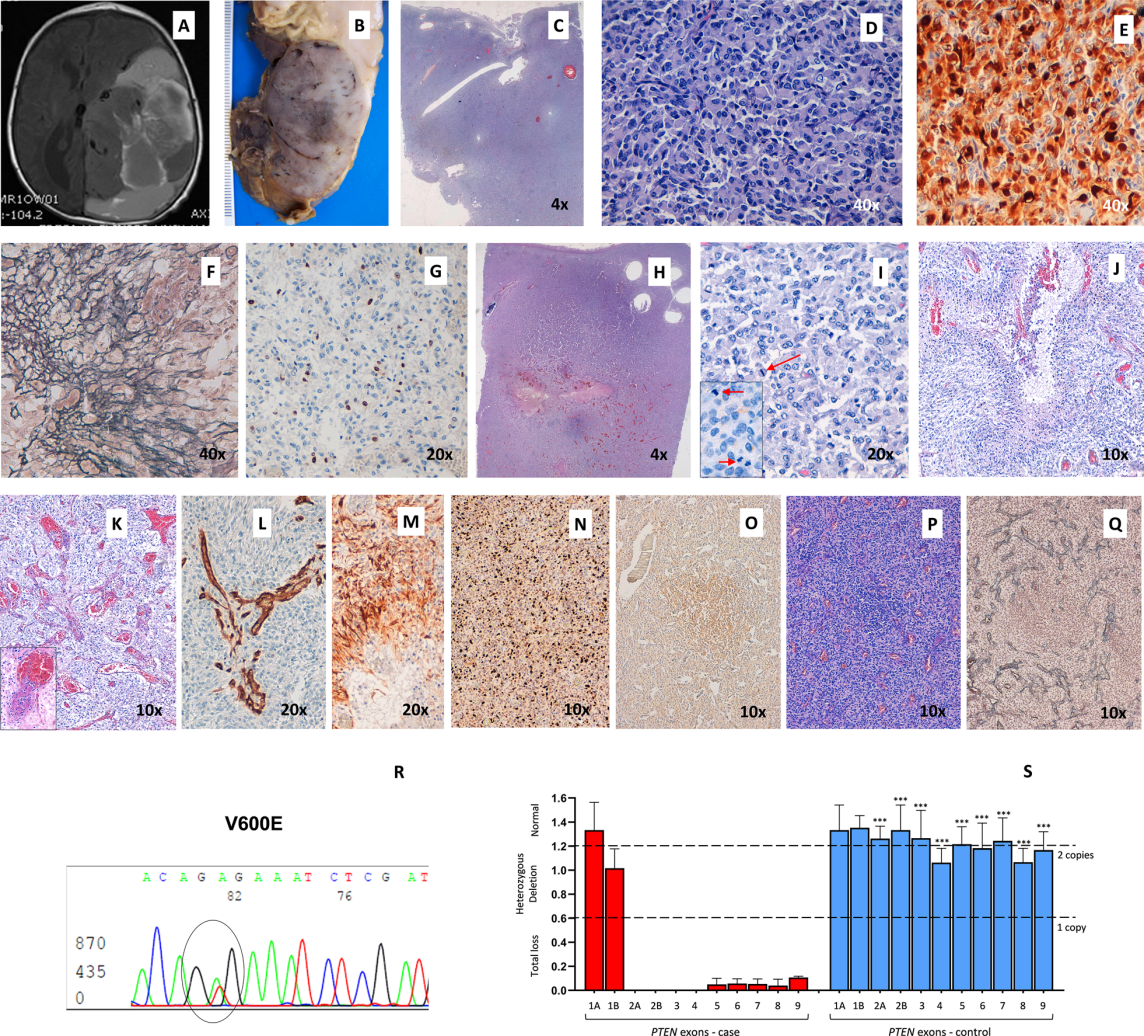
Neuropathological and genetic findings in DIA. Heterogeneous and cystic tumor in a cranial scan (**A**). A well-delineated tumor in the postmortem examination (**B**). Histopathological pattern of DIA: general features (**C**), atypical astrocytes with a gemistocytic pattern (hematoxylin and eosin stain) (**D**), GFAP-positive neoplastic cells (**E**), desmoplastic stroma in Gomori samples (**F**) and low proliferative index (**G**). Histopathological aggressive pattern in DIA: general features (**H**), morphological atypical cells with mitosis (**I**), necrosis with perinecrotic palisading neoplastic cells (**J**), vascular hyperplasia and microthrombosis (**K**), CD34-positive expression in microvascular structures (**L**), GFAP-positive neoplastic cells in perinecrotic areas (**M**), high proliferative index (**N**), area with a small-cell population which express CD133 protein (**O**), that alters the reticulin (**P**) and vessel distribution (**Q**). Molecular alterations: *BRAF V600E* mutation (**R**) and *PTEN* deletion in MLPA analysis (**S**)

The patient was a newborn male, product of the first pregnancy of a 27-year-old mother. Ultrasonography controls during pregnancy reported an adequate gestational age, with a 88 mm biparietal diameter at week 34. However, a subsequent control at week 39 showed a high biparietal diameter, 109 mm and an intracranial hypoechoic image. An emergency caesarean was performed. External analysis showed a cephalic perimeter of 38 cm, cephalic suture softening and prominent fontanels. A MRI scan on day 1 showed a supratentorial tumor of 75 × 63 × 76 mm with heterogenic signal and cystic areas. The tumor produced a midline deviation, hydrocephaly and subfalcian herniation (Fig. 1A). On day 5, surgery was performed. As a result of a hemorrhagic complication, the patient died 24 h later. The tumor was diagnosed as DIA. Postmortem examination of the brain revealed a large, well-delineated tumor in the right parieto-occipital region attached to the dura. Upon section the tumor showed solid areas with a firm texture and a grayish and pale pink color. Residual hemorrhagic areas of the surgical intervention were observed (Fig. 1B).

Histopathological study revealed that the main part of the tumor was composed of uniform atypical astrocytes with moderate pleomorphic nuclei and large eosinophilic cytoplasms. These had a gemistocytic pattern and spindle-shaped cells with benign appearance, forming irregular fascicles or a storiform pattern (Fig. 1C, D). Neoplastic cells showed GFAP expression (Fig. 1E). A fibrillary network of reticulin Gomori-positive fibers completed the morphology (Fig. 1F). A low proliferative index (< 1 mitosis/HPF; < 1% Ki-67 labeling index) (Fig. 1G) without necrosis or vascular endothelial proliferative features was found in these areas. No ganglionic synaptophysin-positive cells were identified. The astrocytic differentiation with heterogeneity in cellular patterns, absence of ganglionic neuronal cells and desmoplastic stroma, support the diagnosis as DIA. However, the internal part of the tumor showed areas with a more aggressive appearance (Fig. 1H, I), with anaplastic GFAP positive cells with cellular polymorphism. Micronecrosis foci (Fig. 1J) and macronecrosis, vascular hyperplasia with endothelial proliferation and microtrombosis (Fig. 1K, L) were observed. An increased proliferative index (11 mitoses/HPF; ≥ 25% Ki-67) (Fig. 1M, N) completes this aggressive histopathological pattern. Finally, the tumor showed a non-desmoplastic area of small embryonal-like cells with low level of differentiation and CD133 expression (Fig. 1O). These cells altered the distribution of the reticulin network and vessels around it (Fig. 1P, Q).

All the genetic studies were performed in the two areas of the tumor, the part with more benign features and the part with an aggressive histological pattern. For the mutational analysis, *BRAF* was amplified by PCR and the products were analysed using the ABI-PRISM 310 Genetic Analyzer automated sequencer. For the study of deletions, multiplex ligation-dependent probe amplification (MLPA) was used (MS–MLPA probe sets P105-C2 and PO44-B1, MRCHolland, The Netherlands). Results showed a *BRAF* mutation that led to a substitution of valine by glutamic acid at position 600 (*V600E*), in both parts of the tumor (Fig. 1R). Moreover, a homozygous deletion in *PTEN* (exons 2 to 9) was found in both areas (Fig. 1S). No changes in *EGFR*, *CDKN2A*, *TP53*, *NFKBIA*, *CDK4*, *MDM2* and *PDGFRA* were found.

## Discussion and conclusions

It is generally accepted that DIA is a low-grade, biologically benign neoplasm [1]. Clinical malignancy is related to the size of the tumor and to a non-successful surgical resection. However, different reports referred to an increase of the proliferative index, histological anaplastic features, endothelial proliferation and necrosis, supporting the existence of an atypical or histologically malignant form of DIA [11, 13–17].

Setty et al. described a case of DIA associated with clusters of malignant cells which expressed GFAP [13]. Darwish et al. also reported a patient with DIA who developed multiple cerebrospinal metastases [14]. Phi and colleagues showed the case of a DIA that recurred eight years after the first surgery and had transformed to overt glioblastoma [15]. Al-Kharazi et al. described a case bearing aggressive clinical and malignant histological features, which continued to grow despite intensive chemotherapy [16]. Narayan et al. reported a case of an infant diagnosed with multifocal, cranial and spinal DIA [17]. In 2018, Wang et al. found a case which recurred ten years after subtotal resection [11]. Finally, we present a case of a DIA with areas with anaplastic GFAP positive cells, necrosis, vascular hyperplasia with endothelial proliferation and increased proliferative index. With this background, it may be concluded that not all tumors with histological features of DIA behave in a benign way, and consequently a close postsurgical follow-up would be required.

Knowledge of genetic alterations in these tumors is limited. Table 1 summarizes the published reports of DIA, indicating whether genetic studies were performed or not. Gessi et al. performed a genome-wide DNA copy number analysis in combination with a multiplex ligation-dependent probe amplification in four DIAs. One case presented focal losses in 17q24 and gains in 1q31.1, and other DIA showed gains in *KDR/PDGFRA*, *MET*, *MDM2* and *BRAF*, but no specific *locus* appeared consistently [18]. From the few genetic features described in DIA, *BRAF V600E* mutation is considered relatively common and the most consistent of all [11, 19]. *BRAF V600E* mutation is involved in different types of tumors of the central nervous system like pleomorphic xanthoastrocytoma, ganglioglioma and extra-cerebellar pilocytic astrocytoma [36], but its relevance in DIA is still controversial. Gessi et al. studied four cases of DIA and described *BRAF V600E* mutation for the first time in this tumor. They found it only in one case and concluded that this mutation was rare in DIA-DIG [18]. Three independent cases of DIA with *BRAF V600E* mutation were published in the next two years, one of them being a non-infantile desmoplastic astrocytoma [10, 19, 20]. Greer et al. published three new cases, all of them negative for the mutation [21]. In 2018, Wang and colleagues examined five DIAs using targeted DNA exome sequencing and found *BRAF V600E* mutations in three of them and *ATRX* and *BCORL1* mutations in one of them, a non-infantile anaplastic tumor [11]. Chaterjee et al. presented two cases of the non-infantile variant of DIA with the canonical *V600E* mutation, describing this mutation as frequent in DIG-DIA tumors [12]. Two more cases were published in 2018, one negative for *BRAF V600E* mutation and the other positive [22, 23]. In 2019, Guerreiro Stucklin et al. established the relevance of *ALK*/*ROS1*/*NTRK*/*MET* alterations in infant gliomas, especially in high-grade gliomas [37]. In 2020, Clarke et al. performed a histologic and genetic study in a collection of 241 gliomas from patients under 4 years of age. Fusions in receptor tyrosine kinase (*RTK*) including those in *ALK*/*ROS1*/*NTRK*/*MET* were found in 21 cases of infantile hemispheric glioma and four DIGs, but in none of the DIA cases. From this series, only three cases were diagnosed as DIAs, without *BRAF* mutations, and one of them with losses in chromosomes 2, 9 and 22, and gains in 5 and 10 [24]. Imperato et al. published in 2021 a cohort of 12 DIA/DIG tumors, where no mutations in *BRAF* were found [25]. Finally, Chiang et al. in 2022, studied separately low-grade and high-grade areas in twelve DIA/DIGs, looking for distinct molecular characteristics [26]. From the twelve cases, only three were DIAs, one of them with the *BRAF V600E* mutation. The study concluded that no recurrent genetic alterations were identified in the series, and high-grade and low-grade areas did not have significant genetic differences that could explain their distinct morphology or biological behavior.

Taken together, although *BRAF V600E* mutation is now considered relatively common in DIA [11, 12, 19], it has been more associated with non-infantile cases of DIA [12], and its presence has been related with the absence of histological features of aggressiveness [11, 12, 36].

In the present work, we show a rare case of a DIA from a newborn, with histological features of malignancy, *BRAF V600E* mutation and a *PTEN* homozygous deletion. Both mutations were present in the different areas of the tumor, apparently showing a lack of correlation between the aggressiveness of the area and the genetic findings. Interestingly, although *PTEN* homozygous deletions are found in high-grade gliomas [38], the present case constitutes the first report of a *PTEN* mutation in a DIA. *PTEN* is a haploinsufficient gene tumor suppressor which regulates various cellular processes, including genomic stability, survival, proliferation and metabolism. Due to its role, a subtle decline or a partial inactivation of PTEN functions substantially induces susceptibility to cancer and tumorigenesis [39, 40]. Several studies suggest that the successive loss of each *PTEN* allele contributes to increase the aggressiveness of gliomas, being involved in the transition from the low to the high grade of these tumors [41–43] and the shortening of median life expectancy and survival [44, 45]. Similarly, the loss of *PTEN* could explain, at least partially, the malignant transformation of a typical benign tumor like DIA. Elucidating the mechanisms underlying tumorigenesis mediated by *PTEN* loss in gliomas would be important and can reveal its potential role in atypical DIAs with this mutation, like the present case.

Since no previous studies reported data about *PTEN* alterations in DIA, it is unknown whereas *PTEN* mutational status may provide useful information for the prognosis of this tumor, and distinguish a new *PTEN*-deficient category of aggressive DIAs. These genetic findings, concurrent with the anaplastic histological characteristics of the tumor, point at the possibility of analyze the mutational status of *PTEN*, together with *BRAF* and other still undiscovered gene mutations in new cases of DIA, in order to better understand the behavior of this rare tumor, provide a new classification of DIAs, achieve a better management of the patients and obtain new targeted molecular therapies.

## Data Availability

All data generated or analysed during this study are included in this published article.

## Abbreviations

*BRAF*: B-Raf gene
DIA: Desmoplastic infantile astrocytoma
DIG: Desmoplastic infantile ganglioglioma
GFAP: Glial fibrillary acidic protein
HPF: High-power field
MLPA: Multiplex ligation-dependent probe amplification
MRI: Magnetic resonance imaging
*PTEN*: Phosphatase and tensin homolog gene
